# Prevalence of Multiple Sensory Deficits in Older Adults in BLSA and ARIC Studies

**DOI:** 10.1093/geroni/igaa057.2921

**Published:** 2020-12-16

**Authors:** Nicole Armstrong, Jennifer Deal, Hang Wang, Jennifer Schrack, Qu Tian, Eleanor Simonsick, Yuri Agrawal, Frank Lin

**Affiliations:** 1 National Institute on Aging, Bethesda, Maryland, United States; 2 Johns Hopkins Bloomberg School of Public Health, Baltimore, Maryland, United States; 3 Johns Hopkins School of Medicine, Baltimore, Maryland, United States; 4 Johns Hopkins University School of Medicine, Baltimore, Maryland, United States

## Abstract

Individual sensory deficits have been associated with adverse outcomes, including dementia, in older adults. Using data from the Baltimore Longitudinal Study of Aging (BLSA) (N=259) and Atherosclerosis Risk in Communities Study (ARIC) (N=962), we examined the prevalence of one, two, or three sensory deficits (hearing, vision, and olfaction) among older adults ≥70 years. Any hearing loss was the most prevalent sensory deficit (70-79 year-olds: 41.3% [BLSA] and 51.2% [ARIC]; ≥80 year-olds: 82.6% [BLSA] and 74.2% [ARIC]), followed by vision loss and olfactory loss. Hearing and vision impairments were more prevalent than hearing and olfactory losses as well as vision and olfactory losses in both age groups and studies There were few people with deficits in all three senses (70-79 year-olds: 3.3% [BLSA] and 2.0% [ARIC]; ≥80 year-olds: 5.8% [BLSA] and 7.4% [ARIC]). Further research should investigate the potential impact of multisensory impairments on older adults.

